# Supplementary material for: Splice-Junction-Based Mapping of Alternative Isoforms in the Human Proteome
Source: Cell Rep. Author manuscript; Available in PMC 2020 Jan 15. (PMC6961840; doi:10.1016/j.celrep.2019.11.026)
Supplement: 2 [file NIHMS1546469-supplement-2.zip › DF1/PXD009737/TestisLysC/TestisLysC_25_PSME4_RSLYRYHSSVENILK.pdf]

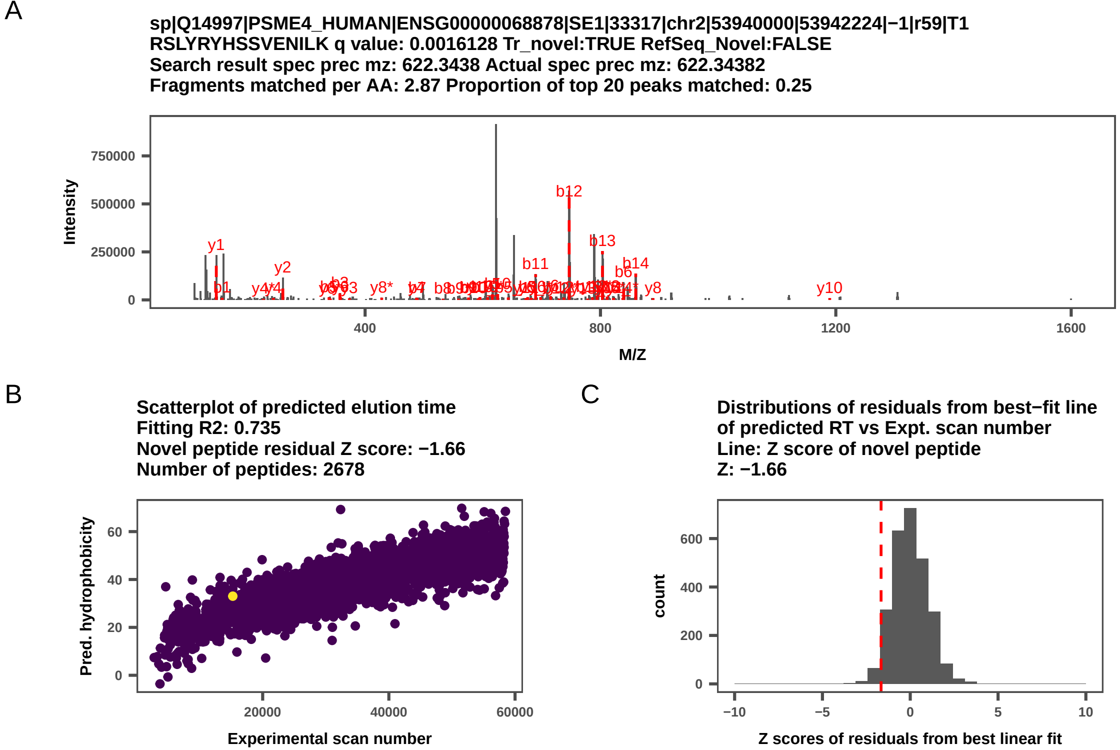
A
sp|Q14997|PSME4_HUMAN|ENSG00000068878|SE1|33317|chr2|53940000|53942224|−1|r59|T1
RSLYRYHSSVENILK q value: 0.0016128 Tr_novel:TRUE RefSeq_Novel:FALSE
Search result spec prec mz: 622.3438 Actual spec prec mz: 622.34382
Fragments matched per AA: 2.87 Proportion of top 20 peaks matched: 0.25
Intensity
750000
500000
250000
0
y1
b1
y2
y8*
b12
b13
b11
b14
b6
y8
y10
400
800
1200
1600
M/Z
B
Scatterplot of predicted elution time
Fitting R2: 0.735
Novel peptide residual Z score: −1.66
Number of peptides: 2678
Pred. hydrophobicity
60
40
20
0
20000
40000
60000
Experimental scan number
C
Distributions of residuals from best−fit line
of predicted RT vs Expt. scan number
Line: Z score of novel peptide
Z: −1.66
count
600
400
200
0
−10
−5
0
5
10
Z scores of residuals from best linear fit
